# Real-Time Traffic Light Detection with Frequency Patterns Using a High-Speed Camera

**DOI:** 10.3390/s20144035

**Published:** 2020-07-20

**Authors:** Kento Yabuuchi, Masahiro Hirano, Taku Senoo, Norimasa Kishi, Masatoshi Ishikawa

**Affiliations:** 1 Department of Creative Informatics, Graduate School of Information Science and Technology, The University of Tokyo, Hongo 7-3-1, Bunkyo-ku, Tokyo 113-8656, Japan; 2 Information Technology Center, The University of Tokyo, Hongo 7-3-1, Bunkyo-ku, Tokyo 113-8656, Japan; hirano@ishikawa-vision.org (M.H.); kishi@ishikawa-vision.org (N.K.); ishikawa@ishikawa-vision.org (M.I.); 3 Graduate School of Advanced Science and Engineering, Hiroshima University, 1-4-1 Kagamiyama, Higashi-Hiroshima City, Hiroshima 739-8527, Japan; taku-senoo@hiroshima-u.ac.jp

**Keywords:** traffic light detection, intelligent vehicles, high-speed camera, image processing, real-time systems

## Abstract

LEDs are widely employed as traffic lights. Because most LED traffic lights are driven by alternative power, they blink at high frequencies, even at twice their frequencies. We propose a method to detect a traffic light from images captured by a high-speed camera that can recognize a blinking traffic light. This technique is robust under various illuminations because it can detect traffic lights by extracting information from the blinking pixels at a specific frequency. The method is composed of six modules, which includes a band-pass filter and a Kalman filter. All the modules run simultaneously to achieve real-time processing and can run at 500 fps for images with a resolution of 800 × 600. This technique was verified on an original dataset captured by a high-speed camera under different illumination conditions such as a sunset or night scene. The recall and accuracy justify the generalization of the proposed detection system. In particular, it can detect traffic lights with a different appearance without tuning parameters and without datasets having to be learned.

## 1. Introduction

Automobiles play an important role in modern society. Modern cars are cheaper, faster, and conbenient to use in many cases; however, many accidents occur every year. Statistics show that 94% of all traffic accidents are due to human error, and approximately, 38,000 vehicle accident deaths are reported each year in the United States [1]. There has been considerable research and development in autonomous vehicle and advanced driver-assistance systems (ADAS), which are expected to predict dangerous events and reduce traffic accidents. However, building automatic driving systems is challenging. An intersection that controls the flow of vehicles and pedestrians is an important aspect of for autonomous driving. In 2017, 890 people died in traffic collisions that involved running a red light in the United States [2]. An automatic driving system can make critical safety decisions in accordance with the state of the traffic lights. Therefore, it should be able to reliably recognize the state of a traffic light from a long distance and in real time. Such an automatic detection system has not been developed yet. As discussed in [3], traffic light detection for complex scenes is a significant challenge. Some of the factors contributing to these complex scenes include various illumination conditions; incomplete shapes due to occlusion; very few pixels for detecting distant traffic lights; and motion blurring due to high-speed driving.

The detection system requires a camera to recognize the state of the traffic light’s lamp pattern. Therefore, many traffic light detection systems are based on image-processing techniques. Numerous methods are based on vision techniques. These can be categorized into three approaches; heuristic model-based approaches, learning-based approaches and auxiliary sensor-based approaches. The heuristic model-based approach uses visual characteristics of a traffic light such as the color and shape [4,5,6,7]. This approach is intuitive, and parameter tuning is easy. The learning-based approach requires many traffic light images to construct a neural net that detects the traffic light. Because of the rapid development of machine learning techniques, this is currently one of the most popular approaches [8,9]. Some leaning based methods include not only traffic light detection but also car detection [8] and approaches that recognize which lane a traffic light belongs to have been developed [10,11]. The auxiliary sensor-based approach uses sensors other than the camera to perform accurate detection by integrating information [12]. Usually, this approach requires a prior map, which has the 3D location of the traffic light and the intersection [13,14]. Because creating a map requires a high cost, it is inconvenient to use it in a large area. Therefore, the scope of this approach is limited.

The appearance of the traffic light varies by country, region, and manufacturer. The different appearances make it difficult for the heuristic model-based and the learning-based approaches to detect it. In contrast, LED traffic lights are widely used because they can achieve better energy efficiency. Because the LED traffic light is driven by an alternate current (AC), it blinks in proportion to the input AC power. LED traffic lights blink at a high frequency, and neither the naked eye nor standard cameras can recognize it. A high-speed camera can capture images at several hundred fps, and it can recognize LED traffic lights. In [15], a hybrid traffic light detection system which combines frequency analysis and visual information with a high-speed camera was proposed. This approach encodes the variation of the brightness for the pixels. Afterwards, it detects the traffic light by extracting the area that shows the specific blinking pattern from the time-series image. By recognizing the blinking traffic light, this approach can achieve more robust detection in comparison to conventional methods that only use visual information.

Although previous work was performed with a high accuracy, it is far from practical use. False detection was observed in scenes that have irregularly reflecting objects or contain blinking self-luminous objects such as electronic bulletin boards. In addition, the conventional method cannot process several hundred images in real-time. This study proposes a real-time traffic light detection method based on blinking LED lights. This system can perform detections more robustly during severe illumination conditions. The contributions of this investigation include the achievement of a robust system with real-time performance that cannot be achieved with the conventional hybrid traffic light detector. The key elements to achieve improved robustness are extracting the blinking with a band-pass filter and state estimation using a Kalman filter. In the proposed method, all processing modules run in a parallel pipeline to enhance the throughput. Therefore, this investigation implemented a detection method with a concise algorithm that does not require a large amount of calculation to achieve real-time processing.

The next section will discuss the related works. The third section describes the proposed traffic light detection method, and the forth section presents the results to verify the performance of this method. Finally, the last section provides the conclusions of this study and our future work.

## 2. Related Works

Many previous studies have focused on visual information. We categorize the traffic light detection methods by the approaches used in previous studies.

### 2.1. Heuristic Model-Based Detection

The heuristic model-based approach uses the color, shape, location and edge information of the traffic light [4,5,7,16]. In some studies, a combination of multiple types of data increased the accuracy of detection [6,17]. The parameters and algorithms are very intuitive and can have a wide range of applications. Some methods that concentrate on the color of traffic lights perform detection by extracting a specific color in the RGB color space. Methods that utilize the HSV color space [6,16] or the LAB color space [4,7] have also been proposed. Meanwhile, some methods that focus on the edge and shape of traffic lights perform detection using Hough transform, which extracts circular regions [17]. In other similar methods, the spot-light detection or radial symmetry transform are used for extracting circular regions [4,17]. In [18], a method was proposed in which multiple cameras with different viewing angles are installed for traffic light detection over a wide range.

### 2.2. Learning-Based Detection

Learning-based approaches have become popular in the recent years owing to the rapid developments in machine learning and object detection techniques [19,20]. In comparison with heuristic model-based approaches, learning-based approaches requires much more training data and computation capacity. However, they are superior to the others owing to their higher robustness to variations and lower tendency to over-fitting. In [21], a method to reduce traffic light candidates based on position and size was proposed. In some detection methods, regions that are not traffic lights are often adopted as candidates. Moreover, these techniques are designed to reduce the computation on the recognition process. It is important to not only detect the traffic lights but also select them corresponding to the vehicle’s current lane for practical application. Some methods detect traffic lights and classify which lane the traffic light corresponds to using CNN [10,11]. In [22], traffic light detection was based on a deep neural net and a prior map. They used a prior map to select traffic lights corresponding to the vehicle’s current lane among the lights detected by the network. By estimating the location of the corresponding traffic lights in the image, the method achieved an efficient reduction of false positives. There are detectors that use a heuristic approach to select the region of interest to enable light weight and real-time detectors while still using CNN recognition [23]. Generally, learning-based approaches require costly network training. However, transfer learning can be used to reduce the computational and time resources during training [24,25]. In some cases, high recognition accuracy is achieved by fusing HOG features and features extracted by CNN [9]. Network models such as RetinaNet [26], and YOLO [8,22] have also been studied. Meanwhile, a method for designing an original background suppression filter and learning filter coefficients using numerous traffic light images without a neural network was proposed [27].

### 2.3. Auxiliary Sensor-Based Detection

The auxiliary sensor-based approach uses additional sensors such as GNSS, accelerometer, gyroscope, stereo-vision and LiDAR. Moreover, in some cases, a smartphone is used as a sensor to detect the traffic lights [12]. Efficient detection is achieved using the IMU and self-location, which help limit the search range in the image. In [13,14], methods that can predict the position of appearance of traffic lights in the camera view based on self-location estimated by LiDAR and GNSS and a prior map are proposed. These studies also proposed an efficient way to create the prior map using a stereo camera. The methods that used auxiliary sensors can detect the traffic light very accurately. However these methods generally require expensive sensors in addition to the cost of creating a prior map.

### 2.4. Hybrid Traffic Light Detection

**Figure 1:**
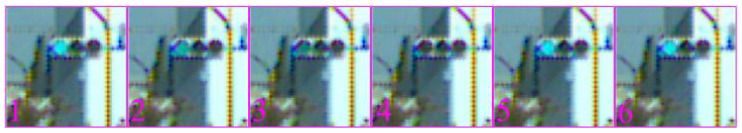
Example of the blinking of LED traffic light observed by a 500 fps camera. The time intervals are 2 ms.

Z. Wu demonstrated the effectiveness of the hybrid approach [15]. The greatest advantage of the hybrid approach is that it detects traffic lights in nighttime when housing can barely be recognized and lights are more difficult to distinguish from other lights. This is because the hybrid approach not only uses visual information but also uses the frequency information. Figure 1 shows the traffic lights driven by a 50-Hz AC taken using a 500 fps camera. This phenomenon is unnoticeable to the naked eye but can be detected by a high-speed camera. Because there are almost no objects blinking as fast as traffic lights, they can be detected by extracting the blinking area. Although previous studies had high accuracy in many scenes, it is still far from practical use for automatic driving systems or driver assistance systems.

This approach is not available in areas where such traffic lights are not installed or on cars that are not equipped with high-speed cameras. However, it is very useful to detect traffic lights at night or in the evening, which is difficult to do by other approaches. The use of a high-speed camera has other advantages. First, flicker problems caused by blinking traffic lights do not need to be addressed. In addition, the images are not blurred even if the vehicle moves at high speed.

## 3. Real-Time Traffic Light Detection System

### 3.1. Overview

**Figure 2:**
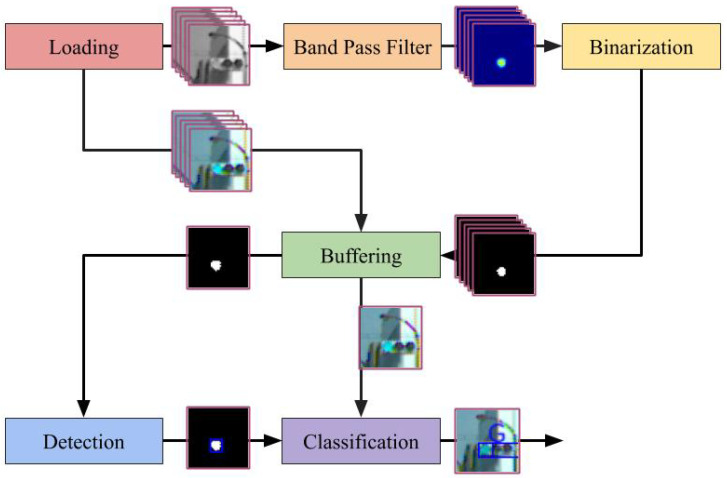
Procedure of the proposed traffic light detection method.

This study considered a case where the traffic light was driven by a 50-Hz AC; thus, the lamp blinks at 100 fps. For this investigation, a high-speed camera that operates at 500 fps was mounted on a car because it could capture five images during a single period of lamp blinking. The proposed detection system consists of six modules that include loading, band-pass filter, binarization, buffering, detection, and classification. The overview of the proposed detection method is provided in Figure 2.

**Figure 3:**
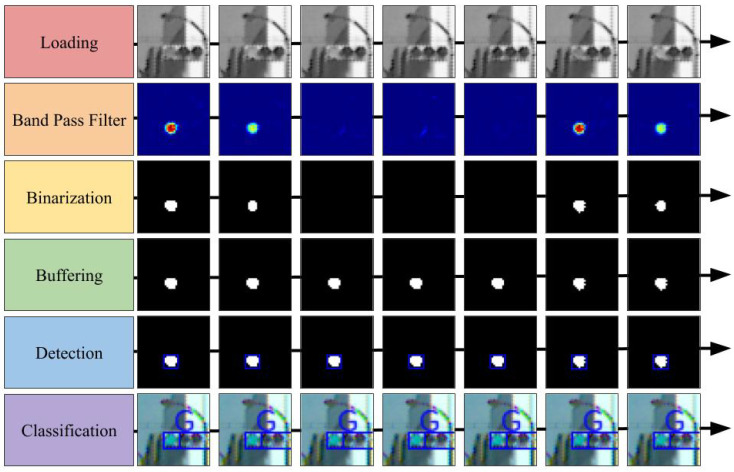
Sequence of the processed images by each module in the system.

The loading module retrieves the images from the camera devices. In the experiment described in the fourth section, the image data were read from a stored video file. To emphasize, this data were not acquired directly from the on-vehicle camera. Moreover, the band-pass filter module applies the band-pass filter to the gray-scaled input image in the frequency domain and not in the spatial domain. This filter enhances the blinking area at a rate of 100 fps. The binarization module first estimates the state of the traffic light dynamics, which includes the blinking amplitude, offset, and phase. It uses the Kalman filter for state estimation. Subsequently, it determines an appropriate threshold for binarizing the filtered image based on the estimated state and finally binarizes the filtered image. The buffering module relays the image, which has stronger signals compared to the previous images. This is because recognizing colors and areas from an image with non-maximum brightness is difficult. The detection module extracts the contours from the peak binarized image. Further, the size and shape are used to exclude candidates to prevent false detections. The classification module classifies the lamp color using the contours and RGB images. A support vector machine (SVM) was used for classification into three classes labeled red, yellow, and green. Figure 3 illustrates a summary of the processed images for each module. As depicted in the figure, the green traffic light was a successfully detected.

### 3.2. Band-Pass Filter Module

This module enhances the area blinking at a specific frequency by applying the band-pass filter to the gray-scaled image over time. We used the IIR filter, which had steep frequency characteristics even in small dimensions, to reduce the amount of computation required for real-time processing. Table 1 shows the conditions for designing the band-pass filter.

An example of the images that were subjected to the filter is provided in the second row of Figure 3.

The band-pass filter module requires more computation than any other modules. Each filtering operation requires addition and multiplication of floating-point numbers in the order of several times the number of pixels. In addition, the operation must be performed several hundred times per second owing to the high-speed camera. If these calculations are performed in a straightforward manner, the real-time performance easily fails. Therefore, we devised several methods to increase the computational speed. In the field of view of an on-vehicle camera, the area in which the traffic light appears on the image is limited. Therefore, we decided not to process the area where no traffic lights would appear. The band-pass filters performed multiplication and summation of floating-point numbers. Consequently, there was negligible data dependency and no conditional branching. Based on this property, we implemented the filtering process by employing parallel processing with single instruction multiple data (SIMD) and OpenMP. In particular, using Intel’s AVX512 instruction set, it was possible to multiply 16 pairs of single-precision floating-point objects simultaneously. By constructing an IIR filter with such instructions, the computation time was significantly reduced.

### 3.3. Binarization Module

**Figure 4:**
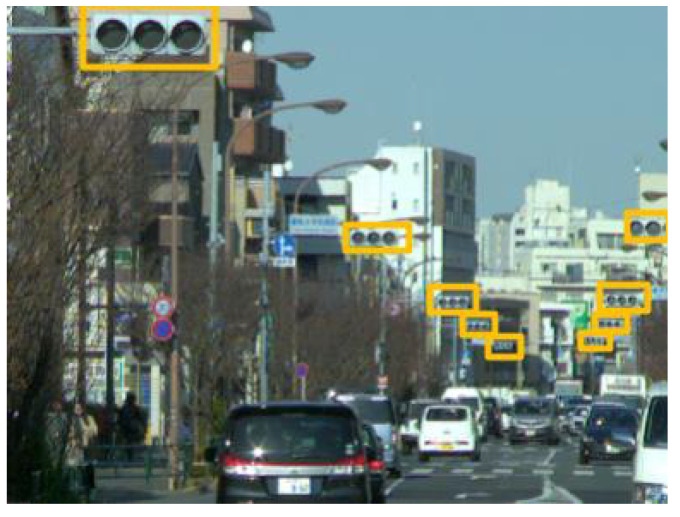
Example of the traffic lights with the same blinking phase.

For the investigation, binarizing the image that was applied to the band-pass filter was necessary to efficiently extract the blinking area. There are two methods of binarization: one is to use a common threshold for the entire image, and the other is to adaptively use the variable threshold for each pixel according to the surrounding pixels. We opted for the first method that used a common threshold as it reduced the computational cost. The appropriate threshold for binarization varies based on the conditions. In particular, the appearance of the traffic light on the image captured during daytime and night differs significantly. The state of traffic light was estimated. It included the amplitude, offset, and phase of the blinking traffic light. Calculating the appropriate threshold removed the disturbance while retaining the traffic lights in the image. For the state estimation, this study assumed that all the traffic lights in an image exist in the same state. This indicates that when there are traffic lights in the image, the blinking amplitude and phase for all traffic lights are the same. Figure 4 shows an example in which the phases of all traffic lights are aligned. In the figure, all the lamps seem to be turned off because all the amplitudes of all lights are the minimum simultaneously.

**Figure 5:**
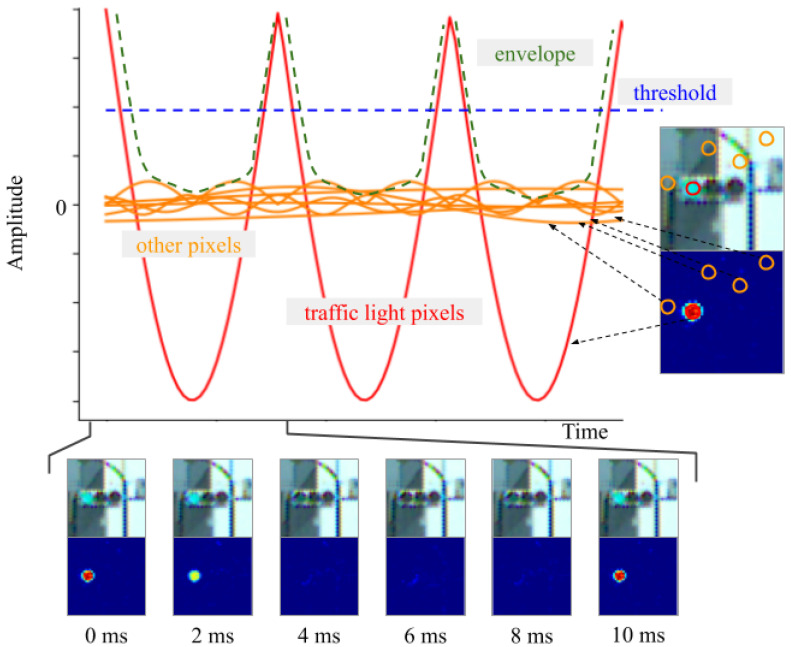
Brightness variation for each pixel.

After the image was passed through the band-pass filter, the pixel value corresponding to the blinking traffic light was obtained based on the absolute value of a sine wave. However, the pixel value in another area was suppressed. The brightness of each pixel in the image was modified, as shown in Figure 5.

In the figure, the red curve represents the pixel corresponding to the traffic light, and the orange curve represents the non-traffic light areas. Here, the envelope of the brightness can be approximated using the following equation:

(1) brightness=A|sin2πft|+b

where, *A* and *b* vary depending on the illumination conditions, and *f* is the frequency of the AC.

When the band-pass filter designed above was applied to videos with 8-bit color depth, *A* ranged from −40 to −100 and *b* ranged from 50 to 130. |A| depends on how sharply the camera captures the blinking traffic lights in contrast to the background. For instance, owing to the dynamic range of the camera, blinking traffic lights in a bright scene do not appear as sharp as they do in a dark scene; therefore, |A| is small. Next, |b| is close to |A|, but |b|−|A| varies depending on blinking disturbances. |b|−|A| represents how strongly blinking noise is retained and this is equal to the length from 0 to the bottom of the envelope in Figure 5. If non-traffic lights are completely removed by a band-pass filter, then |b| and |A| are equal. In contrast, in scenes with many non-traffic lights, such night scenes, there is a large gap between |b| and |A|. Within the same scene, *A* and *b* remain approximately constant over a short time period of 1 s. When binarization was performed using the blue dotted line as the threshold, the noise was removed without affecting the pixels of traffic light signals. An example of the binarized images is shown in the third row of Figure 3. The state was estimated as follows:

(2) $${\left(A,b,\theta =2\pi ft\right)}^{⊤}.$$

Subsequently, the threshold was set using |b|−|A|·k, where *k* is a parameter that adjusts the severity of the threshold. The value of the envelope was obtained each time by searching for the brightest pixel in the image. We approximated the dynamics using a sufficiently simple function; therefore, this study used an extended Kalman filter (EKF) for the state estimation. The process formula of the EKF is as follows:

(3) $$\begin{array}{c} \left(\begin{array}{c} A_{t+1}\\ b_{t+1}\\ \theta_{t+1} \end{array}\right)=\left(\begin{array}{c} A_{t}\\ b_{t}\\ \theta_{t} \end{array}\right)+\left(\begin{array}{c} 0\\ 0\\ 2\pi f\Delta t \end{array}\right). \end{array}$$

The observation formula is given by Equation (1).

We assumed that the process and measurement noise covariances are constant and there is no covariance between any of the variables. Specifically, the covariances of the process noise and the observed noise were expressed as Q and R, respectively, and we defined them as follows:

(4) $$\begin{array}{cc} Q & =\left(\begin{array}{ccc} \sigma_{A}^{2} & 0 & 0\\ 0 & \sigma_{b}^{2} & 0\\ 0 & 0 & \sigma_{\theta }^{2} \end{array}\right) \end{array}$$

(5) $$\begin{array}{cc} R & =\left(\begin{array}{c} \sigma_{\mathrm{brightness}}^{2} \end{array}\right). \end{array}$$

Each self-covariance was adjusted manually. The EKF worked satisfactorily under these loose conditions described above, although more precise values could be set if the characteristics of the band-pass filter and the characteristics of the signal lights were considered. Using the EKF defined above, we estimated the current state of the blinking dynamics of the traffic lights. The threshold was adaptively determined using the estimated state; consequently, the binary image was obtained.

### 3.4. Buffering Module

This module performs two tasks: (1) it seeks the local maximum image from the last images, thus simplifying processing for subsequent modules; and (2) it compensates for the phase delay of the binarized image to aid its synchronization with the RGB image.

#### 3.4.1. Searching for the Local Maximum Image

The blinking of the binary image and the RGB image makes it difficult to classify the color and to detect contours. This module selects an image that is easy to classify from the last five images and passes it to the subsequent modules. Specifically, the last five images are stored using a ring buffer, and the best image is selected using the phase estimated by the EKF in the binarization module.

#### 3.4.2. Compensation for the Phase Delay Caused by the Band-Pass Filter

The binarized image passes through the band-pass filter. Therefore, the signal is delayed with respect to the RGB image. When the camera is moving, the pixel positions of the traffic light in the binary and the RGB images are different. In this case, when classifying the lamp color, the colored traffic light image could be incomplete, because of which its classification may fail. For this investigation, the data transfer of the RGB image was delayed to synchronize it with that of the binarized image. The phase delay owing to the band-pass filter is constant because the blinking frequency of the traffic light and the sampling rate of the camera are invariable. The appropriate delay was calculated in advance and the time consistency was adjusted in the images.

#### 3.4.3. Handling Redundant Calculations Caused by Buffering

The traffic light achieves a maximum brightness once for every five inputs; consequently, the output image of this module is often duplicated. Therefore, the succeeding module uses the same image five times and produces the same output. As a measure to eliminate this redundant processing, we had an option to execute the subsequent modules only once for every five inputs. However, the duplicate output was not omitted to make the system straightforward.

### 3.5. Detection Module

An API called findContours was provided by the open source computer vision library OpenCV to detect the candidate. The contour extracted from the binary image consists of points that cover the periphery of the foreground area on an image. The extracted contours may contain noise that cannot be removed by a band-pass filter or binarization. Consequently, this was filtered using a circularity of the contour, the area, and the position on the image so that they could be removed. Circularity is defined as:

(6) $$\begin{array}{c} \mathrm{circularity}=\frac{4\pi \cdot \mathrm{Area}}{{\left(\mathrm{perimeter}\right)}^{2}}. \end{array}$$

This indicates that a circle has a circularity of 1. That is, circularity indicates how close a contour is to a circle. Considering that the lamp of a traffic light is circular, contours that were not circular were removed.

### 3.6. Classification Module

**Figure 6:**
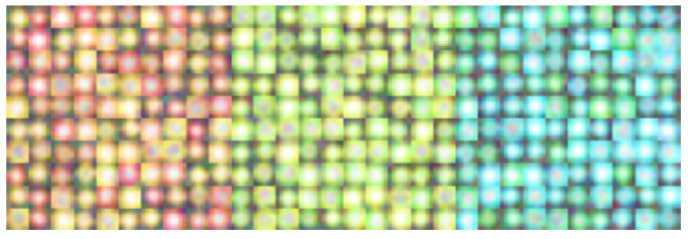
Examples of the training data for the support vector machine.

An SVM was used to recognize the color of the traffic lights. There are three classification labels for SVM: “red”, “yellow” and “green”. The input image for the SVM had a resolution of 10×10 pixels, an 8-bit color-depth, and three channels. When an image was provided as input to the SVM, the bounding box of the contour was transformed into 10×10 pixels. The data for SVM training were generated using random numbers and were not extracted from the actual scene. The reason behind randomly generating images was to make the SVM less dependent on the appearance of the traffic lights. Figure 6 depicts an example of the generated training data. This module is only required to be able to classify three colors. Therefore, the classification in this method was implemented in a very concise manner. Specifically, 100 images were used for each label, and the training was performed using the default parameters of the SVM module in OpenCV.

Traffic lights are always lined up in the order of red, yellow, and green. Based on this, the area of the traffic light housing was also estimated from the color, size, and position of the blinking area.

In Figure 2, there are two paths from buffering and detection to classification. The two paths refer to the data flow of the RGB image and the binarized image, respectively. This system is implemented to reduce the computational complexity and to avoid unnecessary copying of data as much as possible. RGB images are essentially only needed in the classification module, not in the other modules such as the band-pass filter, binarization, and detection. Therefore, the RGB images are passed through the modules in the order of loading, buffering, and classification.

### 3.7. Multi-Thread Processing

**Figure 7:**
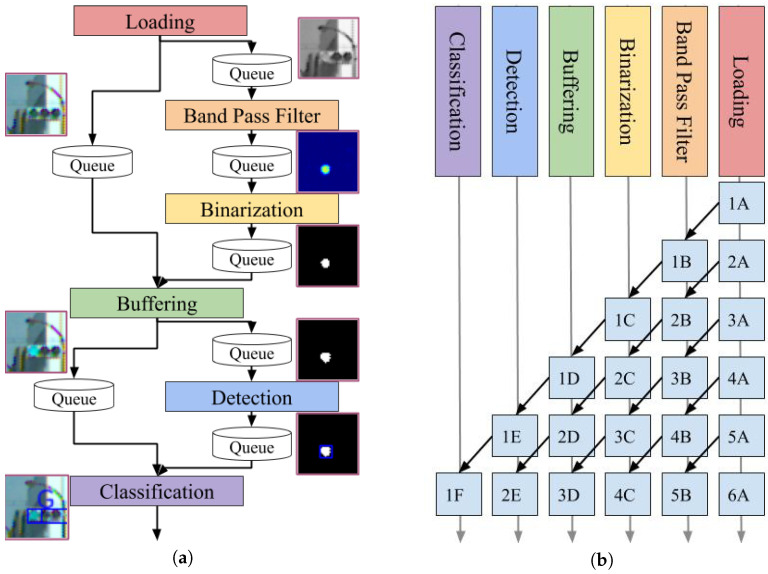
Overview of parallel processing in the system. (**a**) Illustration of the system flowchart with queues. (**b**) Demonstration of the transition of the processed image. The images in the same row were processed simultaneously.

In this system, all modules depend on their previous modules, some of which require considerable computation to process an image. The proposed system must be calculated at 500 fps or more for its practical application. Therefore, this investigation adopted a parallel pipeline process to increase the throughput. Figure 7 provides an overview of the image processing. The modules start the process as soon as the required data are available in the queue. This does not reduce the amount of computation; therefore, the latency either remained constant or it may have been slightly increased owing to the effect of the data transfer between the threads. Moreover, the latency caused by pipeline processing of the six modules can be ignored during the operation because the system processes images at several hundred fps.

## 4. Experimental Results

The video sequences were taken in an urban street in East Japan using a Basler high-speed camera (model: acA800-510uc). It was mounted on a car and its output was an image with 800×600 pixel resolution, and the frame rate was 500 fps. The dataset collection was supported by Kotei Informatics Corporation. In all experiments, considering that the color depth of the camera image is 8 bits, we set the EKF parameters $\sigma_{A}^{2}$ and $\sigma_{b}^{2}$ to 65, $\sigma_{\theta }^{2}$ to 0.03 and $\sigma_{\mathrm{brightness}}^{2}$ to 1600. Data in most public datasets are not captured by high-speed cameras; hence they could not be used to evaluate this system.

### 4.1. Accuracy Evaluation

The accuracy evaluation experiment was performed in four scenes under different times and weather conditions. In each case, the brightness and the contrast between the background and the traffic light were different. The proposed method performed detection with fixed parameters in all scenes. We created a region-wise manually labeled ground truth for the dataset, where each LED traffic light region was represented by a bounding box. This bounding box was assigned to the entire housing of the traffic lights rather than to each lamp. This was because some traffic lights flashed multiple color lamps simultaneously or had arrow-shaped lamps that provide secondary instructions. Therefore, in practical application, it was necessary to recognize not only the lamp of a traffic light but also the entire signal.

Precision and recall were used to evaluate the accuracy, which is defined as

(7) $$\begin{array}{cc} \mathrm{Precision} & =\frac{\mathrm{TP}}{\mathrm{TP}+\mathrm{FP}} \end{array}$$

(8) $$\begin{array}{cc} \mathrm{Recall} & =\frac{\mathrm{TP}}{\mathrm{TP}+\mathrm{FN}} \end{array}$$

where TP, FP and FN indicate true positive, false positive, and false negative, respectively. The detection results were assigned to the ground truth objects and they were determined to be true or false positive by measuring the intersection over union (IoU). The threshold for the IoU was set to 0.4, which is a size that can be popped when the SVM classifies a candidate into the wrong color. This was done considering that in this experiment, we focus on whether the proposed method can detect the lights, and we were not interested in any amount of misalignment.

Table 2 summarizes the detection performance for each scene. The precision and recall values obtained in the daytime exceeded 90%, whereas the recall value was approximately 80% during the sunset and at night, when the detection was difficult. These results show that the proposed method is robust against changes in the illumination environment. We did not measure the accuracy of the color classification of SVM in this dataset because we were interested only in whether the detectors could detect traffic lights. However, if the color is misclassified, the recall values would be lower due to the failure to recognize the housing.

**Figure 8:**
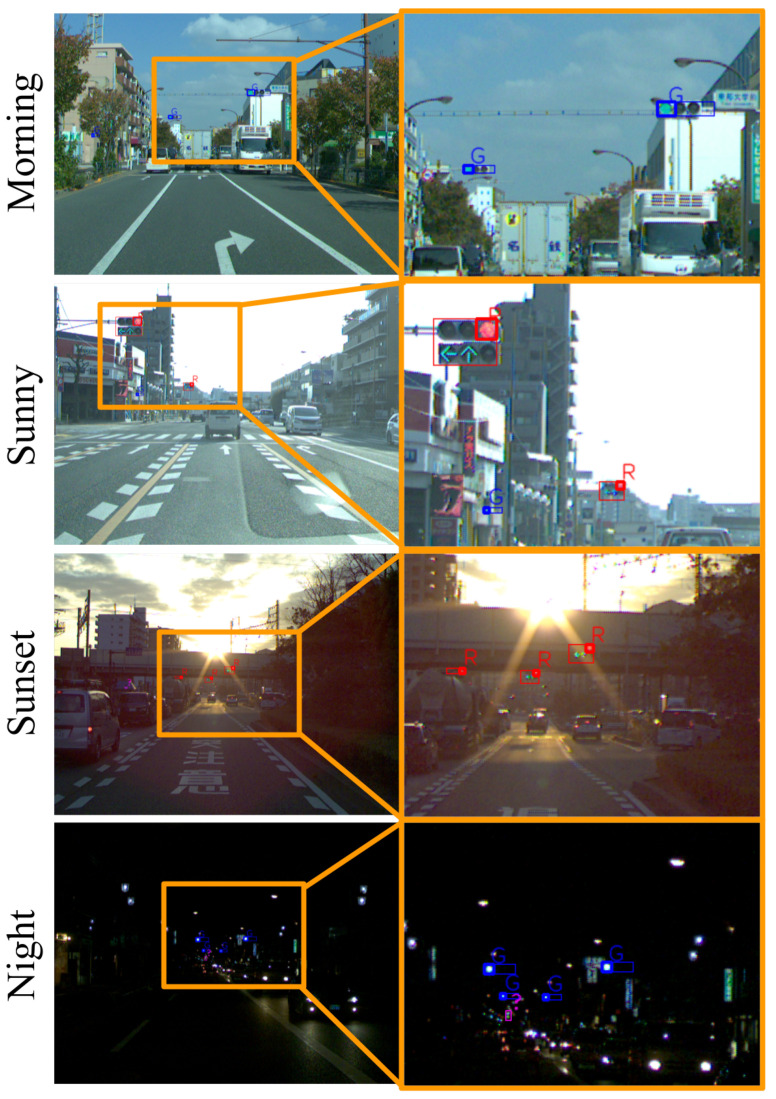
Detection results for all scenes. The left column is the full resolution image. The right column is an enlarged image of the yellow box.

Figure 8 provides the detection results for each scene. A traffic light that appears to be fairly small on the image was successfully detected. Moreover, a traffic light for pedestrians was also detected. In the sunset scene, even a traffic light covered by a smear of sunlight could be detected. In the night scene, the four traffic lights could be correctly recognized despite the confusion caused by streetlights and headlamps. In addition, the traffic light further away could not be recognized as a green lamp; however, they too were detected.

**Figure 9:**
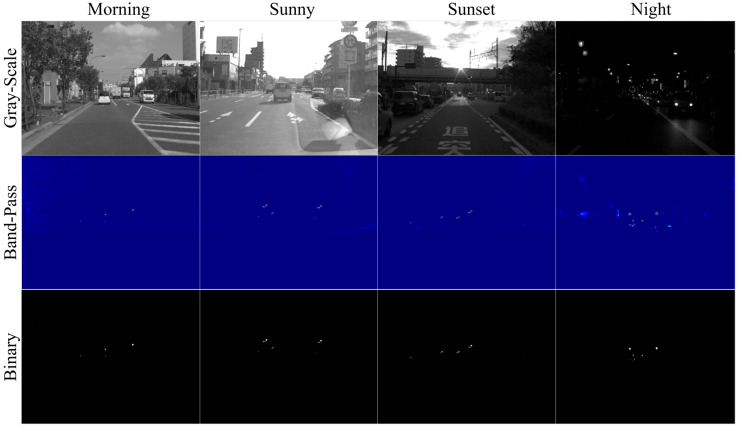
Intermediate results in all scenes. Each column corresponds to a scene. The upper row shows the gray-scaled images, the middle row displays the band-pass-filtered images, and the lower row illustrates the binarized images.

The intermediate results for all scenes are displayed in Figure 9. Some images were passed through the band-pass filter, as shown in the middle row of the figure. It was observed that the area other than the traffic lights is practically suppressed by the filter. In the rightmost center image of the figure, the streetlights and the electronic bulletin board were suppressed. However, in the binary image, they were deducted and only the traffic lights remained. This is because the threshold for binarization was appropriately set, as determined by the state estimation. Thus, this system could detect traffic lights in the same way without the need of parameter adjustment in daytime as well as during the night.

**Figure 10:**
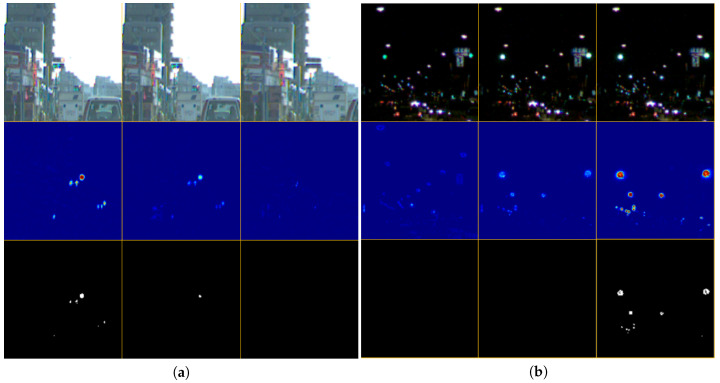
The effects of a blinking disturbance. (**a**) Sunny scene with an electric bulletin board. (**b**) Night scene with many flashing lights, such as streetlights.

The dataset included many blinking lights other than traffic lights. Figure 10 shows images of the two scenes where the effects of disturbances are the most apparent. The sunny scene has an electronic bulletin board blinking at high speed. In the night scene, streetlights and, shop signs are blinking. Most of the disturbances caused by blinking lights are removed by the band-pass filter and binarization. The detection module also contributes to the removal of disturbances. There were not many round displays as bright as traffic lights, blinking at the same height and in the same position as traffic lights, that the proposed system could not distinguish.

However, in the proposed method, detection failed in some cases. First, because this method uses the blinking of a traffic light, the light that is reflected by the cars or buildings may be erroneously detected. This can be resolved by checking the detection position or the size; moreover, it is also possible to reduce the reflected light with a polarizing filter on the lens. Second, the shaking of the camera resulted in occasional failure to extract the blinking area. Because the band-pass filter treats the value of each pixel as an independent signal, it is not possible to obtain blinking on the pixel as the traffic light moved across the pixels frame by frame. Unless the blinking is detected, this system cannot identify traffic lights. In addition, detection may fail if a traffic light whose phase of blinking is shifted, enters the field of view. The phases are generally the same. However, they may be different owing to the differences in the drive circuits.

### 4.2. Comparison with the Conventional Hybrid Detection System

Our dataset taken by a high-speed camera includes many frames where the traffic light is completely off. Therefore, it is not possible to make valid comparisons with detection methods that use other approaches on the same dataset. Other methods that do not consider the blinking of traffic light cannot provide adequate performance. Therefore, we did not compare the accuracy with non-hybrid-based detection methods.

**Figure 11:**
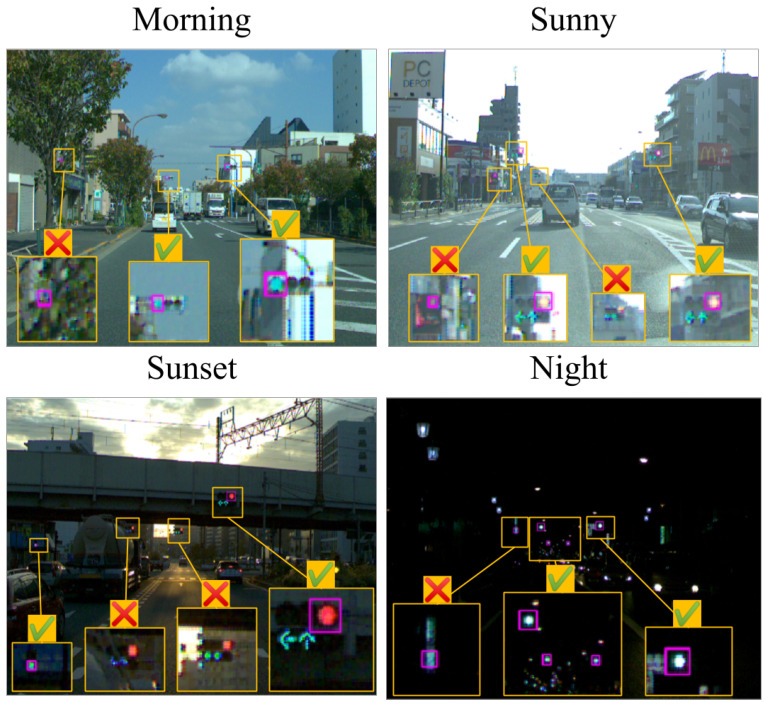
Example of the detection results by the conventional hybrid method. In the morning, the scattering of light by the tree was erroneously detected. When it was sunny, it failed to detect the electronic bulletin board. During the sunset, the traffic light under the viaduct could not be detected. During the night, all the traffic lights were detected; however, the light in a store was incorrectly detected.

In this study, the results were compared with the conventional hybrid detection metho [15] by using a high-speed camera. However, unfortunately, no meaningful comparison can be made between the proposed method and the conventional hybrid-based method. This is because the conventional method only detects circular color lamps and does not perform color classification. Moreover, the method cannot detect the arrow-shaped lamps and entire light housing; thus, it cannot be compared under equal conditions with our proposed method that can detect all of them. Apart from that, the conventional method has limited functionality and is not as practical as the proposed method. From the above, this study analyzed the performance by comparing the output results. The same videos that were used in the accuracy evaluation experiment were used as the input. Figure 11 demonstrates the results of the traffic light detection using the comparison method, which uses the same parameters for detection in all the scenes.

This conventional method uses the blinking of the traffic light for detection; hence, it successfully identified some traffic lights during the day and nighttime. However, there were several false positives and false negatives. From the morning data, the light scattered by the leaves on the street was mistakenly recognized as a blinking traffic light. Similarly, in the case of sunny data, the electric bulletin board, and for the night data, parts of the illumination were mistakenly recognized as traffic light. Moreover, from the sunset data, some traffic lights were not detected even though the traffic lights were close enough.

In the method used for comparison, blinking is encoded based on whether the brightness changes for each pixel are greater than a threshold value. In addition, it employs regions where the time-series pattern of the code matches the specified pattern of traffic light candidates. Therefore, an area where the brightness change happens to match the specified pattern is erroneously recognized as a traffic light, or a traffic light that does not match the pattern due to the disturbance light will not be detected. In particular, there were many false positives because of the scattering of light and the electronic bulletin board. In addition, false negatives occurred when the influence of sunlight was significant.

All these problems with the conventional method were noticeably improved by applying the proposed method. In the proposed method, the band-pass filter was used to extract data corresponding to the blinking area, and the region that was truly blinking represented the signal candidates. Thus, the proposed method can reject instantaneous brightness changes caused by scattering of light. The compared method requires a synchronization process of the blinking cycle every time it searches for an area encoded with a specific pattern. If the synchronization fails, the traffic light detection fails as well; however, in the proposed method, the band-pass filter extract the blinking area without synchronization. Furthermore, the adaptive binarization also contributes to an improvement in the performance. Although the compared method uses pre-calculated specific patterns, its robustness against the illumination change was insufficient because of the changes in brightness patterns that were based on the environment. Meanwhile, the proposed method recognized the blinking dynamics that rarely changed, and the thresholds of the blinking signals were estimated by the Kalman filter. This made it capable of detecting traffic lights in various environments without using specific patterns that may lack robustness.

### 4.3. Efficiency Evaluation

To verify whether this method can be processed in real-time, the calculation time of each module was measured. Because each module is executed in a parallel pipeline, the calculation time of any module must be less than 2 ms to be processed at 500 fps in real-time. The module that displays an image to confirm the detection result was not included in the calculation time because it is not related to traffic light detection. This study measured the computation time for traffic light detection in a 2000-frame video with a resolution of 800×600 pixels. The details of the computer that performed the detection are shown in Table 3. The detection parameters were set to the same values as those used in the detection accuracy evaluation experiment.

The calculation times are summarized in Table 4. Even for the module that takes the longest calculation time, processing was completed in less than 1 ms. In all the modules, the average of the calculation time and the value obtained by adding the standard deviation (SD) to it was less than 2 ms. This indicates that the module has sufficient speed to process a 500-fps video in real-time.

We confirmed that it is possible to run at 500 fps on a laptop PC (Core i7 9750). However, we did not perform detailed experiments on a laptop PC because it would be scarcely capable of performing the required calculations, which should be performed with a margin of computational resources in practical use. The PC used in the evaluation experiments can process the images at enough speed with extra computational power, and the extra resources can be used to conduct other processes. The power consumption of our high-speed camera is 3 W in catalog value. The PC used in the experiments did not include a GPU, and the CPU’s thermal design power (TDP) was 140W. We think they are small enough to be installed in an automated vehicle and can be practically applied.

## 5. Conclusions

This study proposes a real-time traffic light detection method through the recognition of LED blinking caused by AC. A band-pass filter was applied to the input images with the frequency of the AC to efficiently detect the blinking areas. The threshold of the binarization was set adaptively using the state estimated by the Kalman filter. Therefore, the difference in the environments can be detected in a similar manner. Although several previous studies have used visual information for detection such as the appearance of traffic lights, their results vary greatly depending on the illumination. Meanwhile, the proposed method barely depended on this, and it employed more robust frequency information for performing detections during severe conditions. No adjustment of the parameters, such as the heuristic thresholds or neural network training, was required. According to the experimental results, the proposed method performed detection with a high accuracy under various experimental scenes. It was confirmed that all the modules could be processed within 1 ms. Therefore, the proposed method could process more than 1000 fps. A future task is to improve the performance of the classification. On a real road, traffic lights that are not restricted to a particular lane may appear in the angle of view. This requires the ability to properly identify the traffic lights. In addition, the system is required to compensate for the image shaking caused by the vibration of car. If shaking correction can be achieved, the performance of the pixel-by-pixel band-pass filter would improve, and consequently, traffic light detection would become more accurate.

## Figures and Tables

**Table 1:** Condition of the band pass filter.

Parameter	Value
Sampling Rate	500 Hz
Pass Band	95–105 Hz
Filter Type	IIR (Butterworth)
Filter Order	4

**Table 2:** Precision and Recall.

Scene	# of Frame	Precision	Recall
Morning	2000	0.98	0.98
Sunny	2000	0.96	0.91
Sunset	5000	0.89	0.79
Night	3600	0.91	0.84

**Table 3:** PC specifications.

Parameter	Value
CPU	Intel Core i9-7900ZX
Clock	3.30GHz
# of Core (Thread)	10 (20)
Memory	64GB
OS (kernel)	Ubuntu 18.04 (4.15.0-72-generic)
GPU	NO-USED

**Table 4:** Time efficiency for each module.

Module	Average (ms)	SD (ms)	Best (ms)	Worst (ms)
Loading	0.78	0.27	0.39	7.64
Band Pass Filter	0.69	0.37	0.38	3.48
Binarization	0.37	0.08	0.20	0.74
Buffering	0.38	0.18	0.20	2.44
Detection	0.16	0.09	0.06	1.03
Classification	0.02	0.01	0.00	0.25
